# A Personalized Medicine Approach: Psychosocial and Genetic Risk Assessments Predictors of Bariatric Surgery Outcomes After 3 Years

**DOI:** 10.3390/biomedicines14040870

**Published:** 2026-04-10

**Authors:** Panayotis K. Thanos, Shtakshe Chatrath, Colin Hanna, Fiona Comstock, John Butsch, Kenneth Blum, Albert Pinhasov, Lucy Mastrandrea, Teresa Quattrin, Lesley Georger, Alan Posner

**Affiliations:** 1 Behavioral Neuropharmacology and Neuroimaging Laboratory on Addictions, Department of Pharmacology and Toxicology, Clinical Research Institute on Addictions, Jacobs School of Medicine and Biosciences, University at Buffalo, Buffalo, NY 14203, USA; 2 Department of Molecular Biology, Adelson School of Medicine, Ariel University, Ariel 40700, Israelalbertpi@ariel.ac.il (A.P.);; 3 Department of Surgery, Jacobs School of Medicine and Biomedical Sciences, University at Buffalo, Buffalo, NY 14203, USA; 4 Division of Addiction Research & Education, Center for Exercise Sports & Global Mental Health, Western University Health Sciences, Pomona, CA 91766, USA; 5 The Blum Institute of Neurogenetics and Behavior, Austin, TX 78702, USA; 6 Department of Pediatrics, UBMD Pediatrics Division of Endocrinology/Diabetes, University at Buffalo, Buffalo, NY 14203, USA; ldm@buffalo.edu (L.M.);; 7 Department of Biostatistics, University at Buffalo, Buffalo, NY 14203, USA

**Keywords:** metabolic bariatric surgery, substance use disorder, pre-addiction, reward deficiency, hypodomanergia, Roux-en-Y gastric bypass (RYGB), vertical sleeve gastrectomy, personalized medicine

## Abstract

**Background:** This study aimed to further explore the application of genetic risk assessments in 24 metabolic bariatric surgery (MBS) patients to predict weight loss outcomes three years after the procedure. **Methods:** Participants were assessed using the Genetic Addiction Risk Severity (GARS) test, which evaluates neurogenic polymorphisms linked to addiction and reward deficiency. Genetic and psychosocial data collected prior to surgery were analyzed in relation to post-operative weight loss measures, including weight change, body mass index (BMI), percentage of total weight loss (%TWL), and percentage of expected weight loss (%EWL). The analysis examined associations between specific genetic risk alleles, weight-related outcomes at three to four years post-surgery, and psychosocial trait scores. **Results:** Spearman’s correlations revealed that the DRD2 risk allele is negatively correlated with 3-year BMI (r_s_ = −0.481, *p* < 0.05, 95% CI: –0.746 to –0.083). One-way ANOVA indicated that there is a significant difference in 3-year BMI (*p* = 0.018) between 0 and 1 DRD2 risk allele copy. There is also a significant difference in ∆weight (*p* = 0.022), ∆BMI (*p* = 0.014), and %EWL (*p* = 0.032) among the different SNP expression values of the MAOA risk allele. In addition, Spearman’s correlation revealed that FCQ scores are negatively correlated with ∆BMI (r_s_ = −0.470, *p* < 0.05, 95% CI: −0.767, −0.005), %TWL (r_s_ = −0.561, *p* < 0.05, 95% CI: −0.814, −0.129), and %EWL (r_s_ = −0.533, *p* < 0.05, 95% CI: −0.800, −0.090) at 3 years post-surgery and positively correlated with 3-year weight (r_s_ = 0.576, *p* < 0.05, 95% CI: 0.151, 0.821) and 3-year BMI (r_s_ = 0.552, *p* < 0.05, 95% CI: 0.117, 0.810). Lastly, GARS scores are positively correlated with 3-year ∆weight (r_s_ = 0.422, *p* < 0.05, 95% CI: 0.010, 0.712).

## 1. Introduction

Obesity, defined as a BMI greater than 30 kg/m^2^, plays a major role in global comorbidity and mortality [1]. In 2019, over 2 billion adults worldwide were affected by obesity [2]. This growing epidemic shows no signs of slowing down, and if left unaddressed, it is estimated that by 2030, 1.35 billion adults will be overweight and 573 million will be obese. In absolute numbers, this corresponds to 2.16 billion overweight and 1.12 billion individuals with obesity [3].

If untreated, obesity has been shown to be associated with numerous associated medical problems, including Type 2 diabetes mellitus, heart failure, cardiovascular disease, and non-alcoholic fatty liver disease [4].

On average, patients with obesity face 42% higher annual medical costs compared to those of normal weight [5]. Common treatment options for obesity include dietary changes, physical activity, pharmaceutical and nutraceutical therapies, and metabolic bariatric surgery (MBS) [6]. Currently, MBS is considered the most effective intervention for obesity [4]. It is a treatment option indicated for a patient population that fulfills the criteria of either having a BMI ≥ 40 or a BMI ≥ 35, with associated medical problems such as Type II diabetes, cardiovascular disease, hypertension, etc. Laparoscopic sleeve gastrectomy and Roux-en-Y gastric bypass (RYGB) are two commonly performed subtypes of MBS. The laparoscopic sleeve gastrectomy involves the removal of around 75–80% of the stomach, leaving behind a tube-like “sleeve”. The reduction in stomach size limits the amount of food that can be ingested, all the while reducing ghrelin, the hormone responsible for initiating hunger. In a Roux-en-Y, a small pouch is first created from the upper portion of the stomach, considered the “new stomach”, a section that has a capacity of about one ounce. The jejunum of the small intestine is then cut off; one end is attached to the new stomach pouch and the other end is reconnected further down to allow digestive fluids to mix with the food. Any region of the stomach between the pouch and the lower level of small intestine reconnection is considered to be “bypassed”. In one clinical trial of 240 patients, weight loss was 47% and 55% for sleeve gastrectomy and RYGB, respectively, with both procedures leading to improved quality of life [7].

Moreira et al. further examined predictors of weight loss following RYGB. Their study reported significant weight reductions, with average losses of 63% at 12 months and 67% at 24 months in participants with a mean BMI of 53 kg/m^2^. However, they also found that optimal clinical response, defined as a reduction of 50% or more, was more common in those with lower pre-operative weight and greater weight loss at the 12-month follow-up [8].

Individuals with obesity undergoing MBS often exhibit signs of food addiction (FA). A random-effects meta-analysis reported a pre-operative weighted FA prevalence of 32% among bariatric patients, which declined to 15% after surgery [9]. Food addiction is considered a component of Reward Deficiency Syndrome (RDS), a framework used to understand the genetic and epigenetic basis of various addictive behaviors [10]. Gambling, binge eating, and substance use (including alcohol and drugs) are some of the impulsive and compulsive behaviors linked to RDS through their hypodopaminergic effects [11]. Hundreds of studies have investigated the polymorphisms associated with GARS. In a meta-analysis of 74,566 case–control subjects, the DRD2, DRD3, DRD4, DAT1, COMT, OPRM1, and 5HTT polymorphisms were found to be significantly associated with the risk of alcohol use disorder [12].

While MBS is a valuable means in the eradication of obesity, there are post-operative behavioral risk factors associated with these procedures that require further consideration. Newer research has suggested that patients may experience addiction transfer following MBS, in which food addiction is replaced by other RDS behaviors, a few of which including gambling, alcoholism, and drug addiction [13]. The role of various polymorphisms in the risk of addiction transfer furthers the necessity for evaluation of genetic roles in MBS outcomes.

Thanos et al. previously reported genetic data relating to GARS which was compared to MBS outcomes in patients both 6 months and 1 year following MBS [14,15]. Prior to MBS, n = 34 subjects completed a GARS genetic evaluation as well as various psychosocial questionnaires. At 6 months following MBS, various relationships were observed between questionnaire scores and GARS alleles. There was significant correlation between the COMT allele and both EEI (Eating Expectancies Inventory Questionnaire) and PSQI (Pittsburgh Sleep Quality Index) scores, and a positive correlation between the GARBR3 allele and both EEI and FCQ (Food Cravings Questionnaire) scores. GARS analysis in this study showed that at least one copy of each risk allele was present in one or more participants, with the exception of the DRD4, OPRM1, and DAT1 genes, which were not carried by any subjects. Genetic findings revealed a significant correlation between the DRD4 risk allele and both weight change and BMI at 6 months post-MBS. Patients carrying two copies of this allele experienced greater weight changes than those with no copies [14].

At 1 year post-surgery, weight data from n = 30 participants were compared to the baseline GARS results. These findings indicated that MBS had a favorable impact on individuals carrying alleles associated with high addiction risk. Notably, significant weight improvements were observed in participants with one copy of the OPRM1 and DRD2 A1 risk alleles [15].

The present study aimed to build upon these findings to support a personalized medicine approach in optimizing MBS outcomes.

This analysis focused on weight outcomes at 3 years post-surgery, specifically change in weight (∆weight), change in body mass index (∆BMI), and percentage of excess weight loss (%EWL). These outcomes were assessed in relation to risk allele profiles and psychosocial trait scores obtained at baseline, to evaluate their predictive value in long-term post-operative trajectories.

## 2. Methods

### 2.1. Participants

MBS candidates (n = 70) were approached during their final pre-operative consultation at Kaleida Health Weight Management Center in Buffalo, NY, USA. After providing an overall description of the study, 34 candidates (48.6% of those approached) provided informed consent and supplied a cheek swab for genetic analysis. The relatively modest consent rate may reflect participant burden and time constraints, and may introduce potential selection bias.

Notable characteristics of the study participants are as follows: The mean age was 47 years (SD = 12.33); 10.3% were male and 89.7% were female. The average BMI was 43 (SD = 6.02), and the mean pre-operative body weight was 118 kg (SD = 20.76). Participants had an average height of 165 cm (SD = 7.38). Among those who reported race (n = 27), 85.19% identified as White, 11.11% as Black or African, and 3.7% as Hispanic. Pre-operative bloodwork revealed a mean glucose level of 102.62 mg/dL (SD = 31.28), triglycerides of 144.04 mg/dL (SD = 82.45), and cholesterol of 193.2 mg/dL (SD = 38.33).

Exclusion criteria for the study included pregnant individuals, those with significant cognitive or neurological impairments, and incarcerated individuals. Collected data included medical history, associated medical problems, and weight history. Over half of the participants reported a history of childhood obesity. Regarding substance use, 48% reported alcohol consumption (average ≤ 1 drink per week), and one participant reported cigarette use. Additionally, specialists had diagnosed 81% of participants with sleep apnea and 39% with depression, while 42% of the participants reported having orthopedic pain.

**Figure 1:**
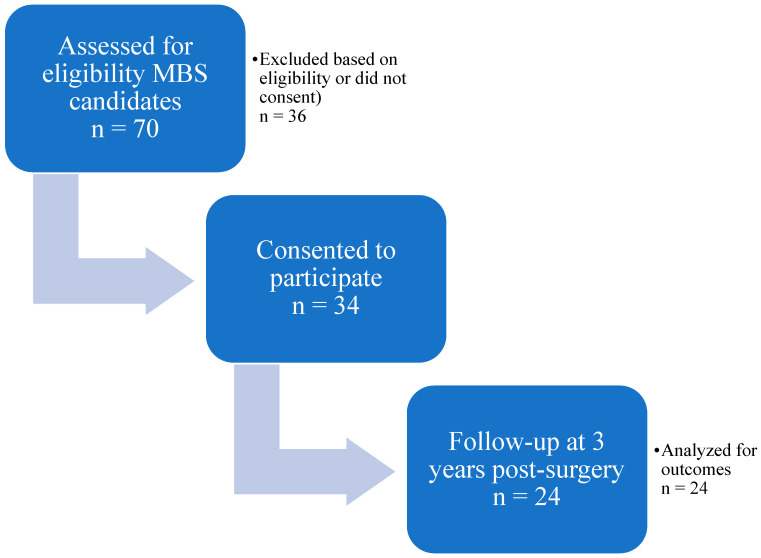
Participant flow diagram for the 3-year follow-up study.

Early in the study, the COVID-19 pandemic caused disruptions to in-person post-operative follow-up, resulting in a smaller sample size than originally anticipated. As a result, sample sizes varied across participants who completed baseline questionnaires, provided GARS samples, and attended follow-up visits at post-operative time points. Follow-up weight data at 3 years post-surgery was available for 24 participants, representing 34% of the originally approached cohort (Figure 1).

### 2.2. Surgery

Of the 24 participants who underwent MBS, 15 received laparoscopic sleeve gastrectomy, while 9 underwent Roux-en-Y gastric bypass.

### 2.3. Data Collection

Health-related parameters before and 3 years after surgery were obtained from electronic health records. Changes in weight and BMI over this period were calculated.

### 2.4. Psychosocial Questionnaires

Participants completed surveys provided in both digital and paper formats. These validated instruments were used to assess psychosocial factors related to obesity and eating behaviors. These surveys were only performed at baseline to serve as a prediction of outcome and were conducted at the patients’ last appointments, 1 week prior to the surgery date. The administered measures included: the Eating Attitudes Test-26 (EAT-26) [16]; Eating Expectancies Inventory (EEI) [17]; modified Yale Food Addiction Scale 2.0 (mYFAS 2.0) [18]; Food Cravings Questionnaire—Trait Reduced (FCQ-TR) [19]; Weight-Influenced Self-Esteem Questionnaire (WISE-Q) [20]; Center for Epidemiologic Studies Depression Scale (CESDS) [21]; Difficulties in Emotion Regulation Scale (DERS) [22]; Chronic Stress Index (CSI) [23]; and the Pittsburgh Sleep Quality Index (PSQI) [24]. This methodology was consistent with previously published approaches [14]. Of the 24 participants with weight data at 3 years post-surgery, most psychosocial measures were available for 19 participants, while PSQI scores were available for only 17 participants. This missing data is likely due to the time-intensive nature of completing the psychosocial questionnaires.

### 2.5. Genetic Addiction Risk Severity (GARS)

The GARS assay is a genetic test that evaluates eleven specific polymorphisms linked to susceptibility for Substance Use Disorder (SUD) and Reward Deficiency Syndrome (RDS). Prior to surgery, cheek swab samples were collected from all 34 participants in the original study cohort and processed following established protocols [25]. DNA was extracted using PCR and analyzed for polymorphisms in the following genes: *DRD1*, *OPRM1*, *DRD2*, *DRD3*, *DRD4*, *COMT*, *DAT1*, *DRD4-R*, *GABRB3*, *HTTLPR*, and *MAOA* [26]. The results were analyzed and reported by Geneus Health (San Antonio, TX, USA) [12,14,15,27,28,29,30]. Individual GARS risk scores were calculated as previously described [26,31,32,33]

### 2.6. Statistical Analysis

All data are presented as mean ± standard deviation (SD) and were analyzed using IBM SPSS Statistics (version 29.0.0), with additional visualization and analyses performed in R (version 4.4.1). Hardy–Weinberg equilibrium was assessed for each locus using an exact test. Normality was assessed using normal probability plots and the Anderson–Darling test. Due to the small sample size, the ordinal or discrete nature of the psychosocial and risk allele variables, and evidence of non-normality in several measures (DERS, EAT-26, and FCQ), Spearman’s rank correlation coefficients were used to examine relationships between psychosocial scores, GARS scores, ∆BMI and ∆weight at 3 years post-surgery. Additional analyses evaluated associations between GARS risk alleles and changes in weight/BMI and psychosocial scores using Spearman’s rank correlation coefficients, and compared mean outcomes across different single nucleotide polymorphism (SNP) expression groups (0, 1, or 2 copies) using analysis of variance (ANOVA). Where applicable, significant ANOVA findings were followed by post hoc comparisons using Tukey’s HSD or Sidak’s test. Individual SNPs were analyzed independently rather than as haplotypes; a formal haplotype inference was not performed, due to the small sample size as discussed above.

### 2.7. Ethics

This study was reviewed and approved by the Institutional Review Board at the University at Buffalo (IRB #IRB00009248) and renewed on 19 March 2025. All participants were fully informed of the study’s purpose and procedures, and provided written informed consent prior to participation.

A total of 70 metabolic bariatric surgery (MBS) candidates were initially assessed for eligibility. Of these, 34 provided informed consent to participate in the study, while 36 declined. Of these, 24 consenting participants completed follow-up assessments at 3 years post-surgery and were included in the final analysis. Six participants from the original 1-year cohort (n = 30) were lost to follow-up before the 3-year assessment. This diagram illustrates participant progression from eligibility assessment to outcome analysis.

## 3. Results

### 3.1. Demographic Results

Initially, 70 MBS candidates were evaluated at the Kaleida Health Weight Management Center in Buffalo, NY. Of these, 34 individuals provided informed consent to participate in the study.

Follow-up data at 3 years post-surgery were available for 24 participants. Among this group, the mean age was 48 years (SD = 12.59); 8.3% were male and 91.7% were female. The average height was 165 cm (SD = 6.16), and the mean pre-operative body weight was 116 kg (SD = 17.33). The mean BMI was 43 (SD = 6.07). Racial distribution showed that 87.5% of participants were White and 12.5% were Black or African. Pre-operative bloodwork was available for a subset of participants and showed the following values: mean glucose = 98.79 mg/dL (SD = 19.29, n = 19); mean triglycerides = 117.89 mg/dL (SD = 42.41, n = 18); and mean cholesterol = 187.56 mg/dL (SD = 34.93, n = 18). Comparisons were made between the 24 patients measured at 3 years to the six participants of the 1-year cohort (n = 30) who were lost to follow-up. There are no significant differences in mean age, weight, BMI, or psychosocial scores at baseline for those lost to follow-up. Also, there are no significant distributional differences in sex or race for those lost to follow-up as compared to those measured at 3 years post-surgery.

### 3.2. Psychosocial and GARS Data

**Figure 2:**
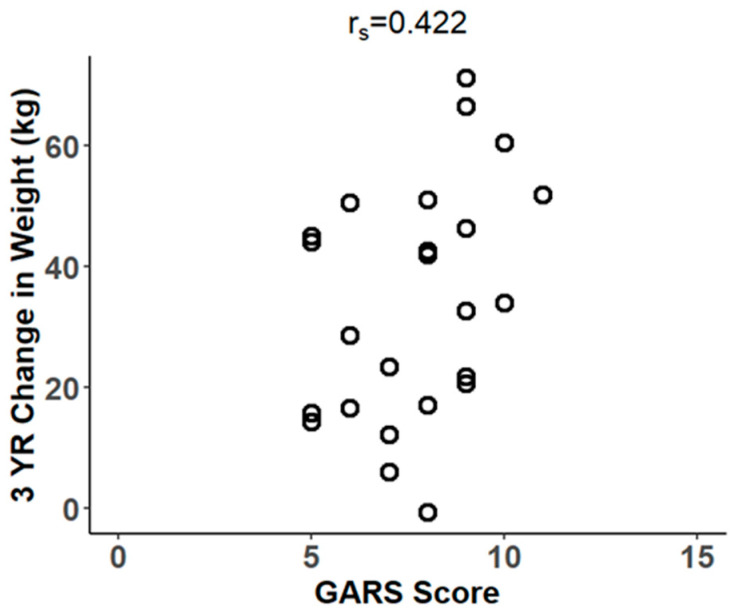
Scatterplot showing the relationship between GARS score and change in body weight (r_s_ = 0.422, *p* < 0.05, 95% CI: 0.010, 0.712) in subjects 3 years post-surgery (n = 24).

GARS scores are positively correlated with 3-year ∆weight (r_s_ = 0.422, *p* < 0.05, 95% CI: 0.010, 0.712) (Figure 2). A GARS score above 7 indicates a high risk for addiction and Reward Deficiency Syndrome (RDS) [15]. Of the 24 subjects who had 3-year follow-up weights, 58.3% (n = 14) had GARS scores above 7. A t-test indicates that subjects with GARS scores above 7 (mean BMI = 28, SD = 4.01) had significantly lower BMI at 3 years post-surgery than subjects with lower GARS scores (mean BMI = 33, SD = 4.81) (*p* < 0.05, 95% CI for the difference in mean BMI: −8.47, −0.99). There are no significant associations between having a GARS score above 7 and weight, ∆weight, ∆BMI, or %EWL at 3 years post-MBS, which differs from results found at 1 year post-MBS.

**Figure 3:**
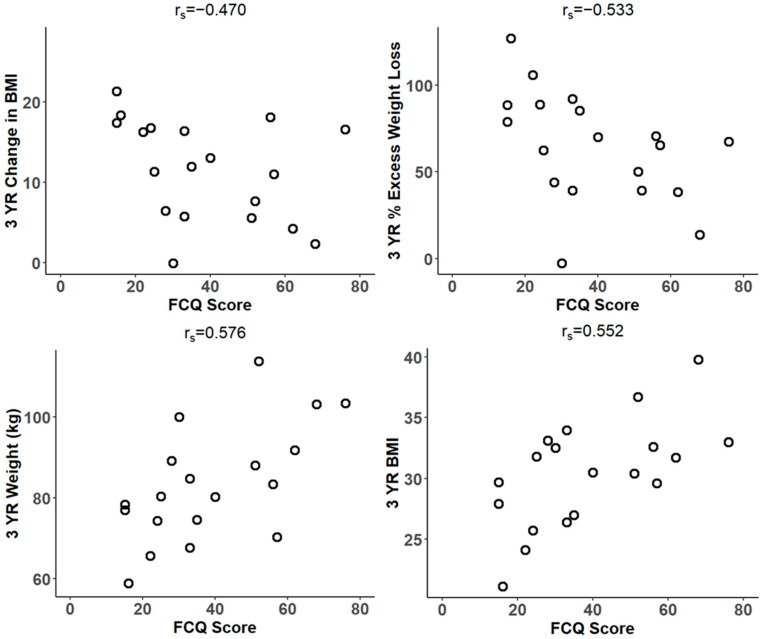
Scatterplots showing the relationship between FCQ score and change in BMI (r_s_ = −0.470, *p* < 0.05, 95%, CI: −0.767, −0.005), percent excess weight loss (r_s_ = −0.533, *p* < 0.05, 95% CI: −0.800, −0.090), body weight (r_s_ = 0.576, *p* < 0.05, 95% CI: 0.151, 0.821), and BMI (r_s_ = 0.552, *p* < 0.05, 95% CI: 0.117, 0.810) in subjects 3 years post-surgery (n = 19).

Spearman’s correlation (r_s_) revealed that FCQ scores are negatively correlated with ∆BMI (r_s_ = −0.470, *p* < 0.05, 95%, CI: −0.767, −0.005), %TWL (r_s_ = −0.561, *p* < 0.05, 95% CI: −0.814, −0.129), and %EWL (r_s_ = −0.533, *p* < 0.05, 95% CI: −0.800, −0.090) at 3 years post-surgery. They are positively correlated with 3-year weight (r_s_ = 0.576, *p* < 0.05, 95% CI: 0.151, 0.821) and 3-year BMI (r_s_ = 0.552, *p* < 0.05, 95% CI: 0.117, 0.810) (Figure 3).

A summary of the survey findings is presented in Table 1.

### 3.3. Risk Allele Correlations

All loci were assessed using an exact test and showed no evidence of deviation from Hardy–Weinberg equilibrium (all *p* > 0.05).

**Figure 4:**
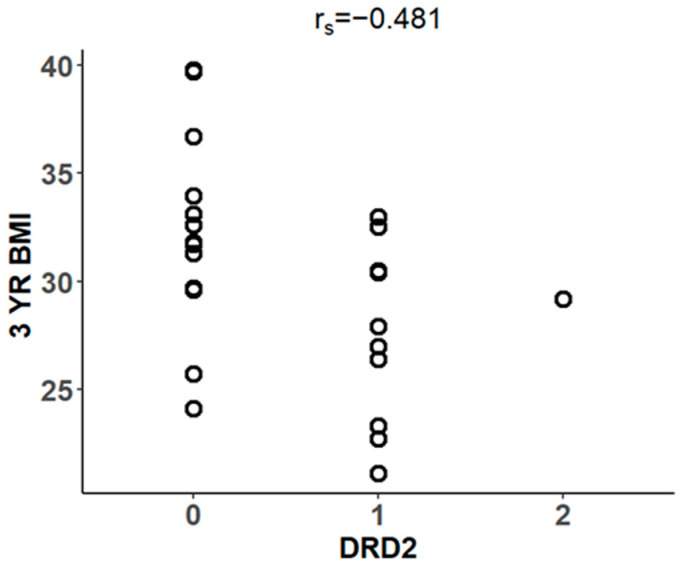
Scatterplot showing the relationship between DRD2 risk allele and BMI (r_s_ = −0.481, *p* < 0.05, 95% CI: –0.746 to –0.083) in subjects 3 years post-surgery (n = 24).

Spearman’s correlation analysis revealed that the DRD2 risk allele was negatively associated with 3-year BMI (r_s_ = –0.481, *p* < 0.05, 95% CI: –0.746 to –0.083) (Figure 4). However, no significant correlations were observed between the DRD2 allele and 3-year weight, ∆weight, ∆BMI, or %EWL, which contrasts with findings reported at the 1-year post-MBS mark.

**Figure 5:**
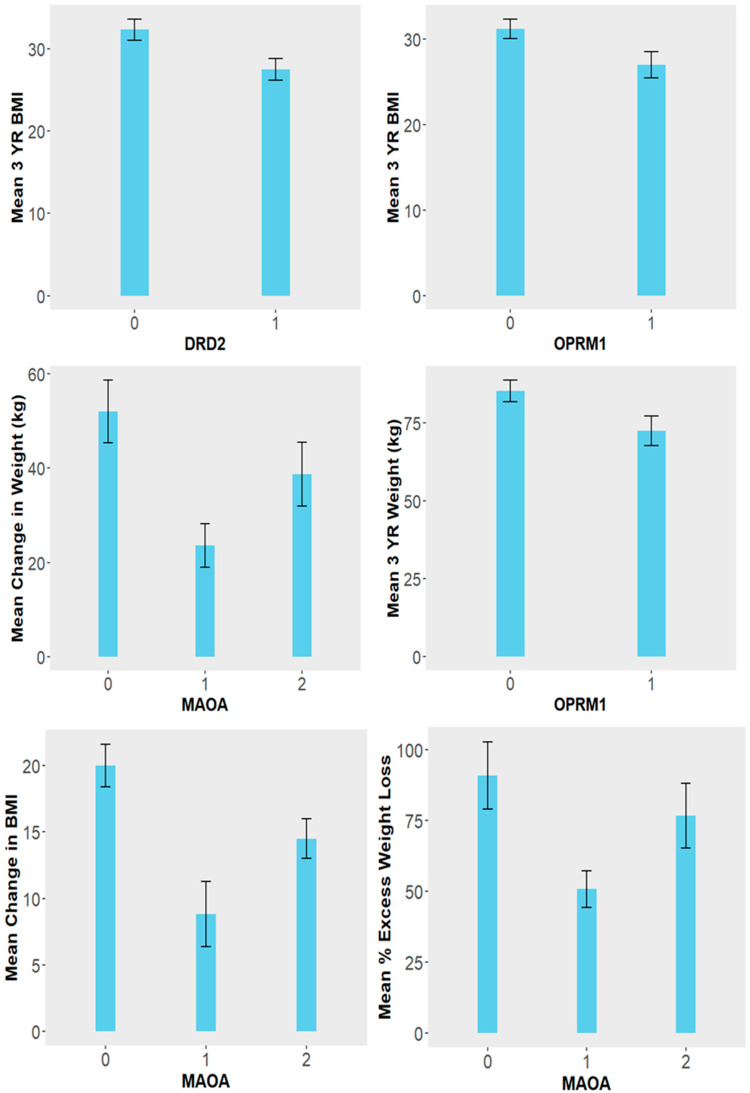
Mean weight loss metrics by number of risk alleles (0, 1, or 2) for comparisons with statistically significant group differences. Error bars represent ± standard error of the mean (SEM). Significant differences between groups were determined by one-way ANOVA with Tukey’s post hoc test (*p* < 0.05).

A one-way ANOVA was conducted to compare the means of weight, ∆weight, ∆BMI, %TWL, and %EWL across different SNP expression groups (zero, one, or two copies). A significant difference in 3-year BMI (*p* = 0.018) was found between individuals with 0 and 1 copy of the DRD2 risk allele. A borderline significant difference was also observed for 3-year BMI (*p* = 0.064) and 3-year weight (*p* = 0.069) between individuals with zero and one copy of the OPRM1 risk allele. For the MAOA risk allele, significant differences were found across SNP expression values for ∆weight (*p* = 0.022), ∆BMI (*p* = 0.014), %TWL (*p* = 0.013), and %EWL (*p* = 0.032). Tukey’s HSD post hoc analysis showed that these differences were significant between individuals with zero and one copy of the MAOA risk allele for ∆weight (*p* = 0.025), ∆BMI (*p* = 0.015), and %EWL (*p* = 0.050), but not between those with zero and two copies or one and two copies (Figure 5). Subjects with one copy of the MAOA risk allele have a significantly lower %TWL than subjects with zero or two copies.

Subjects with two copies of the OPRM1 risk allele were not represented in the sample. Only one subject had two copies of the DRD2 risk allele and was therefore excluded from group comparisons. This individual had a 3-year weight of 85.6 kg and BMI of 29.2, with a ∆weight of 21.9 kg and ∆BMI of 7.9 over the follow-up period.

## 4. Discussion

Consistent with findings at 6 months and 1 year post-MBS, the results of this study demonstrate a favorable response to MBS among individuals with higher addiction risk. Participants with elevated GARS scores (scores > 7) exhibited greater changes in weight. ANOVA results showed borderline significant differences between individuals with zero and one copy of the MAOA risk allele, with those carrying one copy displaying lower average weight, BMI, and %EWL. In addition, both ANOVA and Spearman’s correlation analyses revealed a negative association between 3-year BMI and the presence of one copy of the DRD2 risk allele. However, no significant correlations were observed between ∆weight, ∆BMI, or %EWL at 3 years.

The DRD2 gene is located on chromosome 11q23, and its A1 risk allele has been widely associated with various substance and non-substance addictions [34,35,36,37,38,39,40,41,42,43]. Carriers of the DRD2 A1 allele exhibit reduced D2 dopamine receptor availability [44,45], which may result in D2 receptor super-sensitivity [40]. This neurobiological profile has been linked to increased severity of alcoholism [12,46], obesity [47], and addiction relapse [45].

The presence of the DRD2 A1 allele has also been associated with several characteristics of obesity [48,49,50]. Notably, significant correlations have been found between the presence of the DRD2 allele and BMI in individuals seeking weight loss treatment [35]. The A1 obesity phenotype has been linked to parental obesity, post-pubertal onset, and a preference for carbohydrate-rich foods [40]. Moreover, the A1 allele was identified in 64.3% of individuals with a strong preference for carbohydrates and in 45.2% of 73 individuals with obesity who did not abuse alcohol or drugs [51].

Given these associations, one possible interpretation of our findings is that D2 modulation may contribute to the differential responses to the metabolic and behavioral changes following MBS. It may seem contradictory that BMI values following MBS are benefitted by a polymorphism coding for addictive eating behaviors and obesity. Despite this relationship, further correlations have been observed within the DRD2 A1 allele, including increased compliance to weight loss programs and alcoholism addiction treatment [52]. The A1 phenotype has been associated with decreased body weight, BMI, and fat mass among subjects following resistance training and calorie restriction of weight loss [53]. Further, alcoholics who are carriers of the A1 allele demonstrated optimal attenuations in craving and anxiety when undergoing dopamine agonist therapy for alcohol addiction (Bromocriptine treatment) [52].

One hypothesis is that individuals with dopaminergic variants linked to reward dysregulation may experience alterations in reward processing following bariatric surgery that influence eating behavior or adherence to post-operative dietary changes. D2 dopamine receptor availability is significantly decreased in individuals with severe obesity [5]. In parallel, D2 availability is known to decrease with overstimulation from overeating [54,55,56] suggesting that the wanting mechanism was reduced in the patients with obesity as the surgery bypassed D2 super sensitivity.

However, the present study was not designed to test this mechanism directly, and these interpretations should be considered hypothesis-generating. Prospective studies incorporating neurobiological and behavioral measures will be necessary to determine whether dopaminergic signaling pathways, including potential D2 receptor adaptations, contribute to differential weight loss responses after MBS.

The results of this study revealed a borderline significant difference in mean BMI and mean weight 3 years following MBS between patients with zero and one copy of the OPRM1 risk allele. These correlations were not seen at 6 months or 1 year following MBS. Further, presence of the OPRM1 allele was seen to be positively correlated with Difficulties in Emotion Regulation Scale (DERS) scores among participants at 6 months and 3 years following MBS (psychosocial questionnaires were not administered at 1 year post-MBS).

The OPRM1 allele has been seen to be associated with feeding behavior, fat intake, obesity, and amygdala volume in a study of 598 adolescents [57,58]. This is consistent with existing knowledge that the Mu–Opioid receptor plays a role in motivation, hedonic behaviors, and reward processing [59]. BOLD fMRI data has revealed that the OPRM1 allele is associated with greater amygdala volume. Amygdala volume is negatively correlated with fat intake [57].

Further studies have analyzed the availability of OPRM1 in individuals with obesity. In one study utilizing [(11)C]carfentanil PET scans, BMI was negatively correlated with OPRM1 availability, and 13 women with Class III obesity (BMI ≥40 kg/m^2^) were observed to have decreased OPRM1 availability in several brain regions, including the ventral striatum, insula, and thalamus [59]. Brain responses to palatable food cues have been observed via BOLD fMRI and include activation in various regions including the amygdala, ventral striatum, and hypothalamus. These reward responses were negatively correlated with OPRM1 availability [58].

Another notable gene, MAOA, encodes enzymes responsible for the breakdown of monoamine neurotransmitters, including dopamine, norepinephrine, and serotonin [60,61]. Variations in the MAOA gene can therefore influence dopamine levels specifically [62], and have been associated with several psychiatric conditions, such as substance use disorders, conduct disorder, and antisocial personality disorder [60,61,63].

The role of this gene in obesity and psychiatric traits warrants further investigation. One study examining MAOA and COMT genotypes in individuals with obesity compared to controls found no significant association between MAOA genotype and obesity status [64]. Conversely, another study examining the same gene and repeat sequences relevant to our research (3.5R, 4R) identified a significant relationship between MAOA genotype and both body weight and BMI [61]. Additionally, research involving young Portuguese adults reported a correlation between body fat and the MAOA 3R genotype, but only among male participants [29].

In our study, three years post-MBS, we observed a significant difference in mean changes in weight, BMI, and %EWL between individuals with zero and one copy of the MAOA risk allele. However, no significant differences were found between those with zero and two copies or between one and two copies. These findings may be attributed to subtle variations in dopamine regulation associated with this genotype.

Regarding the psychosocial data, patients’ Gene Addiction Risk Scale (GARS) scores were assessed at baseline. As previously noted, the GARS test evaluates genetic predisposition to addictive behaviors, with a score above 7 indicating an elevated risk for addiction. Among the 24 patients who completed the 3-year follow-up, 58.3% had GARS scores above 7. Notably, these patients demonstrated significantly lower BMI at 3 years post-surgery compared to those with lower GARS scores.

Patients were also followed up with the Food Cravings Questionnaire at the 3-year follow-up. Analysis of the data revealed that FCQ scores were negatively correlated with changes in BMI and % EWL at 3 years post-surgery and positively correlated with weight and BMI at 3 years. Thus, there is evidence to suggest that as FCQ scores increase, patients tend to experience less weight loss or greater weight following MBS. Specifically, a higher FCQ score is linked to a smaller reduction in BMI or less weight loss relative to the patient’s excess weight, suggesting that higher food cravings or problematic eating behaviors may hinder optimal clinical response after MBS. Furthermore, higher FCQ scores are linked to higher weight and BMI three years post-surgery, meaning that patients reporting higher levels of food cravings or problematic eating behaviors (as measured by FCQ) tend to experience more recurrent weight gain and higher BMI after surgery.

In addition, GARS scores were found to be positively correlated with 3-year ∆weight, at 3 years post-surgery. As a high GARS score reflects a greater genetic or psychological predisposition to addictive behaviors or difficulties in managing reward-related cravings, patients with high GARS scores may indeed struggle to maintain weight loss due to challenges related to food cravings or imbalance with regard to their reward system, as suggested by a smaller reduction in their BMI and more weight gain over the 3-year period post-surgery. With a greater difficulty in sustaining optimal clinical response, there is an increased likelihood of recurrent weight gain and achieving a higher BMI over time.

Several limitations should be considered when interpreting these findings. The sample size was relatively small (n = 24), which increases the risk of both Type I and Type II errors, limits statistical power, and may reduce the generalizability of the results. Furthermore, given the small sample size and the exploratory nature of the study, analyses did not adjust for potential confounders. In addition, although many correlations were tested, adjustments for multiple comparisons were not made, in order to maintain consistency with the analysis of data collected at earlier timepoints (6 months and 1 year). This also increases the risk of Type I errors. Additionally, the observed pattern of missingness in the psychosocial scores is likely due to the burden associated with completing lengthy questionnaires. This suggests that the missing completely at random (MCAR) assumption may not hold, as the probability of missingness may be related to participant characteristics such as time availability or fatigue. In addition, the cohort included participants from multiple self-reported racial and ethnic backgrounds (White, Black/African American, and Hispanic). Due to the limited sample size, formal assessment of population stratification (e.g., principal component analysis or genomic control) was not conducted. Population stratification is a recognized source of confounding in genetic association studies, as differences in allele frequencies across ancestry groups may influence observed associations. Therefore, residual confounding due to population stratification cannot be excluded and should be considered when interpreting the genetic findings of this study. Finally, the majority of participants underwent sleeve gastrectomy rather than Roux-en-Y gastric bypass. Consequently, our study did not have sufficient data to directly compare neurohormonal or metabolic outcomes between these two bariatric surgery subtypes, which limits interpretability regarding procedure-specific effects.

## 5. Conclusions

This study provides novel evidence that genetic predispositions linked to addiction and reward processing significantly influence long-term outcomes following MBS. Specifically, individuals with elevated GARS scores, which indicate a higher genetic risk for addictive behaviors, were found to exhibit more favorable weight loss outcomes at 3 years post-surgery.

Notably, the presence of specific alleles, including DRD2 A1, MAOA, and OPRM, was associated with significant differences in BMI, weight loss, and excess weight loss (%EWL), suggesting that genetic factors may influence not only the physiological outcomes but also the behavioral responses to MBS.

Furthermore, the integration of psychosocial measures such as the Difficulties in Emotion Regulation Scale (DERS), Food Cravings Questionnaire (FCQ), and GARS scores underscores the complex interplay between genetic predispositions, craving-related behaviors, and optimal clinical response.

These findings challenge the conventional assumption that addiction-related genotypes consistently predict poor surgical outcomes. Instead, they suggest that certain alleles may enhance sensitivity to the neurochemical and behavioral changes induced by bariatric procedures, potentially facilitating more favorable responses. Clinically, these insights could inform more personalized treatment approaches, enabling healthcare providers to offer targeted guidance and support tailored to an individual’s genetic and psychological profile.

However, the results of this study should be considered preliminary, given the modest sample size and single-center design. Replication in independent, larger cohorts will be essential to confirm these associations and support their clinical utility. Clinically, these insights may eventually inform more personalized treatment approaches, enabling healthcare providers to offer guidance tailored to an individual’s genetic and psychological profile.

This report is the third part of a longitudinal study observing the genetic and psychosocial effects of MBS outcomes. Future studies will track these same data at longer time intervals post-MBS. Notes of recurrent weight gain, including for substance and non-substance addictive behaviors, will be closely monitored as well. These subjects will continue to be monitored for long-term outcomes beyond the present study.

## Figures and Tables

**Table 1:** Summary of findings: Mean and standard deviations of body weight measures and genetic and psychosocial correlations of body weight data after MBS at 3 years post-operation.

	Mean ± SD
BMI	30.2 ± 4.9
∆BMI	13.0 ± 7.3
∆Weight	34.5 ± 19.5 kg
% Excess Weight Loss (EWL)	67.3 ± 30.7%
% Total Weight Loss (TWL)	28.3 ± 14.5
	Significant Correlations (*p* < 0.05) with the Following Variables
Genetic Addiction Risk Severity (GARS) Score	∆Weight
GARS Score	BMI at 3 Years
(Categorized as ≤ 7 or > 7)	
Food Cravings Questionnaire (FCQ) Scores	Weight at 3 Years
	BMI at 3 Years
	∆BMI
	%EWL
	%TWL
*DRD2* Risk Allele	BMI at 3 Years

## Data Availability

The datasets presented in this article are not readily available because the data are part of an ongoing study. Requests to access the datasets should be directed to corresponding author.

## References

[1] Mohammadian Khonsari N., Khashayar P., Shahrestanaki E., Kelishadi R., Mohammadpoor Nami S., Heidari-Beni M., Esmaeili Abdar Z., Tabatabaei-Malazy O., Qorbani M. (2022). Normal Weight Obesity and Cardiometabolic Risk Factors: A Systematic Review and Meta-Analysis. Front. Endocrinol..

[2] Caballero B. (2019). Humans against Obesity: Who Will Win?. Adv. Nutr..

[3] Kelly T., Yang W., Chen C.S., Reynolds K., He J. (2008). Global burden of obesity in 2005 and projections to 2030. Int. J. Obes..

[4] Kloock S., Ziegler C.G., Dischinger U. (2023). Obesity and its comorbidities, current treatment options and future perspectives: Challenging bariatric surgery?. Pharmacol. Ther..

[5] Apovian C.M. (2016). Obesity: Definition, comorbidities, causes, and burden. Am. J. Manag. Care.

[6] Bray G.A., Frühbeck G., Ryan D.H., Wilding J.P. (2016). Management of obesity. Lancet.

[7] Grönroos S., Helmiö M., Juuti A., Tiusanen R., Hurme S., Löyttyniemi E., Ovaska J., Leivonen M., Peromaa-Haavisto P., Mäklin S. (2021). Effect of Laparoscopic Sleeve Gastrectomy vs. Roux-en-Y Gastric Bypass on Weight Loss and Quality of Life at 7 Years in Patients with Morbid Obesity: The SLEEVEPASS Randomized Clinical Trial. JAMA Surg..

[8] Moreira S.H.C., Alvarez-Leite J.I., Souza R.P., Resmini G.C., Resende C.M.M., de Marco L., Bastos-Rodrigues L. (2024). Predictors of Successful Weight Loss in Extremely Obese Individuals Undergoing Roux-en-Y Gastric Bypass Surgery. J. Obes. Metab. Syndr..

[9] Praxedes D.R., Silva-Júnior A.E., Macena M.L., Gearhardt A.N., Bueno N.B. (2023). Prevalence of food addiction among patients undergoing metabolic/bariatric surgery: A systematic review and meta-analysis. Obes. Rev..

[10] Blum K., Thanos P.K., Wang G.J., Febo M., Demetrovics Z., Modestino E.J., Braverman E.R., Baron D., Badgaiyan R.D., Gold M.S. (2018). The Food and Drug Addiction Epidemic: Targeting Dopamine Homeostasis. Curr. Pharm. Des..

[11] Gondré-Lewis M.C., Bassey R., Blum K. (2020). Pre-clinical models of reward deficiency syndrome: A behavioral octopus. Neurosci. Biobehav. Rev..

[12] Blum K., Han D., Gupta A., Baron D., Braverman E.R., Dennen C.A., Kazmi S., Llanos-Gomez L., Badgaiyan R.D., Elman I. (2022). Statistical Validation of Risk Alleles in Genetic Addiction Risk Severity (GARS) Test: Early Identification of Risk for Alcohol Use Disorder (AUD) in 74,566 Case-Control Subjects. J. Pers. Med..

[13] Blum K., Bailey J., Gonzalez A.M., Oscar-Berman M., Liu Y., Giordano J., Braverman E., Gold M. (2011). Neuro-Genetics of Reward Deficiency Syndrome (RDS) as the Root Cause of “Addiction Transfer”: A New Phenomenon Common after Bariatric Surgery. J. Genet. Syndr. Gene Ther..

[14] Thanos P.K., Hanna C., Mihalkovic A., Hoffman A.B., Posner A.R., Busch J., Smith C., Badgaiyan R.D., Blum K., Baron D. (2023). The First Exploratory Personalized Medicine Approach to Improve Bariatric Surgery Outcomes Utilizing Psychosocial and Genetic Risk Assessments: Encouraging Clinical Research. J. Pers. Med..

[15] Thanos P.K., Hanna C., Mihalkovic A., Hoffman A., Posner A., Butsch J., Blum K., Georger L., Mastrandrea L.D., Quattrin T. (2023). Genetic Correlates as a Predictor of Bariatric Surgery Outcomes after 1 Year. Biomedicines.

[16] Garner D.M., Olmsted M.P., Bohr Y., Garfinkel P.E. (1982). The eating attitudes test: Psychometric features and clinical correlates. Psychol. Med..

[17] Fitzsimmons-Craft E.E., Keatts D.A., Bardone-Cone A.M. (2013). Eating Expectancies in Relation to Eating Disorder Recovery. Cogn. Ther. Res..

[18] Koball A.M., Borgert A.J., Kallies K.J., Grothe K., Ames G., Gearhardt A.N. (2021). Validation of the Yale Food Addiction Scale 2.0 in Patients Seeking Bariatric Surgery. Obes. Surg..

[19] Meule A., Hermann T., Kübler A. (2014). A short version of the Food Cravings Questionnaire-Trait: The FCQ-T-reduced. Front. Psychol..

[20] Trottier K., McFarlane T., Olmsted M.P., McCabe R.E. (2013). The Weight Influenced Self-Esteem Questionnaire (WISE-Q): Factor structure and psychometric properties. Body Image.

[21] Smarr K.L., Keefer A.L. (2011). Measures of depression and depressive symptoms: Beck Depression Inventory-II (BDI-II), Center for Epidemiologic Studies Depression Scale (CES-D), Geriatric Depression Scale (GDS), Hospital Anxiety and Depression Scale (HADS), and Patient Health Questionnaire-9 (PHQ-9). Arthritis Care Res..

[22] Mancinelli E., Cottu M., Salcuni S. (2024). Validation of the Difficulties in Emotion Regulation Scale-Short Form in a sample of Italian adolescents. J. Clin. Psychol..

[23] Schulz P., Jansen L.J., Schlotz W. (2005). Stressreaktivität: Theoretisches Konzept und Messung. Diagnostica.

[24] Buysse D.J., Reynolds C.F., Monk T.H., Berman S.R., Kupfer D.J. (1989). The Pittsburgh Sleep Quality Index: A new instrument for psychiatric practice and research. Psychiatry Res..

[25] Blum K., Chen A.L.C., Thanos P.K., Febo M., Demetrovics Z., Dushaj K., Kovoor A., Baron D., Smith D.E., Roy A.K. (2018). Genetic addiction risk score (GARS)™, a predictor of vulnerability to opioid dependence. Front. Biosci..

[26] Blum K., Kazmi S., Modestino E.J., Downs B.W., Bagchi D., Baron D., McLaughlin T., Green R., Jalali R., Thanos P.K. (2021). A Novel Precision Approach to Overcome the “Addiction Pandemic” by Incorporating Genetic Addiction Risk Severity (GARS) and Dopamine Homeostasis Restoration. J. Pers. Med..

[27] Blum K., Bowirrat A., Baron D., Lott L., Ponce J.V., Brewer R., Siwicki D., Boyett B., Gondre-Lewis M.C., Smith D.E. (2020). Biotechnical development of genetic addiction risk score (GARS) and selective evidence for inclusion of polymorphic allelic risk in substance use disorder (SUD). J. Syst. Integr. Neurosci..

[28] Blum K., Modestino E.J., Gondre-Lewis M., Chapman E.J., Neary J., Siwicki D., Baron D., Hauser M., Smith D.E., Roy A.K. (2018). The Benefits of Genetic Addiction Risk Score (GARS(™)) Testing in Substance Use Disorder (SUD). Int. J. Genom. Data Min..

[29] Moran M., Blum K., Ponce J.V., Lott L., Gondré-Lewis M.C., Badgaiyan S., Brewer R., Downs B.W., Fynman P., Weingarten A. (2021). High Genetic Addiction Risk Score (GARS) in Chronically Prescribed Severe Chronic Opioid Probands Attending Multi-pain Clinics: An Open Clinical Pilot Trial. Mol. Neurobiol..

[30] Toups M.S., Myers A.K., Wisniewski S.R., Kurian B., Morris D.W., Rush A.J., Fava M., Trivedi M.H. (2013). Relationship between obesity and depression: Characteristics and treatment outcomes with antidepressant medication. Psychosom. Med..

[31] Glatt S.J., Faraone S.V., Lasky-Su J.A., Kanazawa T., Hwu H.G., Tsuang M.T. (2009). Family-based association testing strongly implicates DRD2 as a risk gene for schizophrenia in Han Chinese from Taiwan. Mol. Psychiatry.

[32] Carpenter C.L., Wong A.M., Li Z., Noble E.P., Heber D. (2013). Association of dopamine D2 receptor and leptin receptor genes with clinically severe obesity. Obesity.

[33] Cameron J.D., Riou M., Tesson F., Goldfield G.S., Rabasa-Lhoret R., Brochu M., Doucet É. (2013). The TaqIA RFLP is associated with attenuated intervention-induced body weight loss and increased carbohydrate intake in post-menopausal obese women. Appetite.

[34] Crist R.C., Reiner B.C., Berrettini W.H. (2019). A review of opioid addiction genetics. Curr. Opin. Psychol..

[35] Sanwald S., Montag C., Kiefer M. (2022). Cumulative Genetic Score of DRD2 Polymorphisms Is Associated with Impulsivity and Masked Semantic Priming. J. Mol. Neurosci..

[36] Niu Y.M., Zhang J., Tang H., Cao L.H., Jiang T.Y., Hu Y.Y. (2023). Association between DRD2/ANKK1 rs1800497 C > T polymorphism and post-traumatic stress disorder susceptibility: A multivariate meta-analysis. Front. Neurosci..

[37] Tsou C.C., Chou H.W., Ho P.S., Kuo S.C., Chen C.Y., Huang C.C., Liang C.S., Lu R.B., Huang S.Y. (2019). DRD2 and ANKK1 genes associate with late-onset heroin dependence in men. World J. Biol. Psychiatry.

[38] D’Ambrosio E., Pergola G., Pardiñas A.F., Dahoun T., Veronese M., Sportelli L., Taurisano P., Griffiths K., Jauhar S., Rogdaki M. (2022). A polygenic score indexing a DRD2-related co-expression network is associated with striatal dopamine function. Sci. Rep..

[39] Blum K., Sheridan P.J., Wood R.C., Braverman E.R., Chen T.J., Comings D.E. (1995). Dopamine D2 receptor gene variants: Association and linkage studies in impulsive-addictive-compulsive behaviour. Pharmacogenetics.

[40] Ribeiro G., Maia A., Cotovio G., Oliveira F.P.M., Costa D.C., Oliveira-Maia A.J. (2023). Striatal dopamine D2-like receptors availability in obesity and its modulation by bariatric surgery: A systematic review and meta-analysis. Sci. Rep..

[41] Nakamura Y., Koike S. (2024). Daily fat intake is associated with basolateral amygdala response to high-calorie food cues and appetite for high-calorie food. Nutr. Neurosci..

[42] Arinami T., Itokawa M., Aoki J., Shibuya H., Ookubo Y., Iwawaki A., Ota K., Shimizu H., Hamaguchi H., Toru M. (1996). Further association study on dopamine D2 receptor variant S311C in schizophrenia and affective disorders. Am. J. Med. Genet..

[43] Noble E.P., Blum K., Ritchie T., Montgomery A., Sheridan P.J. (1991). Allelic association of the D2 dopamine receptor gene with receptor-binding characteristics in alcoholism. Arch. Gen. Psychiatry.

[44] Noble E.P. (1998). The D2 dopamine receptor gene: A review of association studies in alcoholism and phenotypes. Alcohol.

[45] Uhl G., Blum K., Noble E., Smith S. (1993). Substance abuse vulnerability and D2 receptor genes. Trends Neurosci..

[46] Ferreira C.M., Reis N.D.D., Castro A.O., Höfelmann D.A., Kodaira K., Silva M.T., Galvao T.F. (2021). Prevalence of childhood obesity in Brazil: Systematic review and meta-analysis. J. Pediatr..

[47] Cai N., Choi K.W., Fried E.I. (2020). Reviewing the genetics of heterogeneity in depression: Operationalizations, manifestations and etiologies. Hum. Mol. Genet..

[48] Nonino C.B., Barato M., Ferreira F.C., Delfino H.B.P., Noronha N.Y., Nicoletti C.F., Junior W.S., Welendorf C.R., Souza D.R.S., Ferreira-Julio M.A. (2022). DRD2 and BDNF polymorphisms are associated with binge eating disorder in patients with weight regain after bariatric surgery. Eat. Weight Disord..

[49] Zhu J.F., Chen L.H., Yuan K., Liang L., Wang C.L. (2018). Dopamine receptor D2 polymorphism is associated with alleviation of obesity after 8-year follow-up: A retrospective cohort study in obese Chinese children and adolescents. J. Zhejiang Univ. Sci. B.

[50] Volkow N.D., Wang G.J., Fowler J.S., Telang F. (2008). Overlapping neuronal circuits in addiction and obesity: Evidence of systems pathology. Philos. Trans. R. Soc. Lond. B Biol. Sci..

[51] Volkow N.D., Wang G.J., Baler R.D. (2011). Reward, dopamine and the control of food intake: Implications for obesity. Trends Cogn. Sci..

[52] Haghighi A., Melka M.G., Bernard M., Abrahamowicz M., Leonard G.T., Richer L., Perron M., Veillette S., Xu C.J., Greenwood C.M. (2014). Opioid receptor mu 1 gene, fat intake and obesity in adolescence. Mol. Psychiatry.

[53] Nummenmaa L., Saanijoki T., Tuominen L., Hirvonen J., Tuulari J.J., Nuutila P., Kalliokoski K. (2018). μ-opioid receptor system mediates reward processing in humans. Nat. Commun..

[54] Karlsson H.K., Tuominen L., Tuulari J.J., Hirvonen J., Parkkola R., Helin S., Salminen P., Nuutila P., Nummenmaa L. (2015). Obesity is associated with decreased μ-opioid but unaltered dopamine D2 receptor availability in the brain. J. Neurosci..

[55] Need A.C., Ahmadi K.R., Spector T.D., Goldstein D.B. (2006). Obesity is associated with genetic variants that alter dopamine availability. Ann. Hum. Genet..

[56] Ziegler C., Domschke K. (2018). Epigenetic signature of MAOA and MAOB genes in mental disorders. J. Neural Transm..

[57] Chmurzynska A., Mlodzik-Czyzewska M.A., Radziejewska A., Wiebe D.J. (2021). Hedonic Hunger Is Associated with Intake of Certain High-Fat Food Types and BMI in 20- to 40-Year-Old Adults. J. Nutr..

[58] Avsar O., Kuskucu A., Sancak S., Genc E. (2017). Are dopaminergic genotypes risk factors for eating behavior and obesity in adults?. Neurosci. Lett..

[59] Kanarik M., Grimm O., Mota N.R., Reif A., Harro J. (2022). ADHD co-morbidities: A review of implication of gene × environment effects with dopamine-related genes. Neurosci. Biobehav. Rev..

[60] Dias H., Muc M., Padez C., Manco L. (2016). Association of polymorphisms in 5-HTT (SLC6A4) and MAOA genes with measures of obesity in young adults of Portuguese origin. Arch. Physiol. Biochem..

[61] Manco L., Machado-Rodrigues A.M., Padez C. (2022). Association study of common functional genetic polymorphisms in SLC6A4 (5-HTT) and MAOA genes with obesity in portuguese children. Arch. Physiol. Biochem..

[62] Bosun A., Albu-Kalinovic R., Neda-Stepan O., Bosun I., Farcas S.S., Enatescu V.R., Andreescu N.I. (2024). Dopaminergic Epistases in Schizophrenia. Brain Sci..

[63] Jiang Z., Chen Z., Chen X. (2023). Candidate gene-environment interactions in substance abuse: A systematic review. PLoS ONE.

[64] Heidinger B.A., Cameron J.D., Vaillancourt R., De Lisio M., Ngu M., Tasca G.A., Chyurlia L., Doucet É., Doucette S., Maria Obregón Rivas A. (2021). No association between dopaminergic polymorphisms and response to treatment of binge-eating disorder. Gene.

